# The causal effect of triglyceride and high blood pressure on IgA nephropathy: a Mendelian randomization study

**DOI:** 10.3389/fmed.2024.1338462

**Published:** 2024-02-08

**Authors:** Yijun Yang, Yang Li, Xinshun Feng, Chenguang Ding, Jing Zhang, Zunwei Liu

**Affiliations:** ^1^First Affiliated Hospital of Xi’an Jiaotong University, Xi’an, China; ^2^Department of Renal Transplantation, Nephropathy Hospital, The First Affiliated Hospital of Xi’an Jiaotong University, Xi’an, China

**Keywords:** immunoglobulin A nephropathy, high blood pressure, triglyceride, Mendelian randomization, causality, enrichment analysis

## Abstract

**Background:**

It has been reported that high blood pressure (HBP) and triglyceride (TG) are considered risk factors in immunoglobulin A nephropathy (IgAN). This study aimed to explore the causalities between HBP and TG, and IgAN on the basis of Mendelian randomization (MR) analysis.

**Methods:**

Firstly, the genome-wide association study (GWAS) summary data of IgAN (GCST90018866) and two exposure factors, TG (ukb-d-30870_raw) and HBP (ukb-a-437), were sourced from the GWAS Catalog and Integrative Epidemiology Unit (IEU) OpenGWAS databases, respectively. In this study, five methods were utilized to perform MR analysis after picking out single nucleotide polymorphisms (SNPs) as instrumental variables, including MR-Egger, weighted median, simple mode, weighted mode, and inverse variance weighted (IVW), followed by the sensitivity analysis containing the heterogeneity, horizontal pleiotropy test and leave-one-out (LOO) analysis. Finally, the enrichment analysis and interaction network construction of genes corresponding to SNPs of HBP and TG were performed.

**Results:**

The univariate MR results revealed that HBP and TG regarded as risk factors were causally related to IgAN [TG: *p* = 0.046, odds ratio (OR) = 1.065, 95% confidence interval (CI) = 1.001–1.133; HBP: *p* = 7.09 × 10^−7^, OR = 1.970, 95% CI = 1.507–2.575] based on random-effect IVM method, of which TG had a weaker impact. The reliability of these univariate MR results was certified by the sensitivity analysis, in which there was no horizontal pleiotropy and exaggerated influence of each SNP. Furthermore, HBP was markedly causally related to IgAN (*p* = 0.000512) with the help of multivariate MR analysis, rather than TG (*p* = 0.332). Therefore, when HBP and TG occur simultaneously, HBP is a direct influencing factor on IgAN. Ultimately, a total of 208 and 153 genes separately corresponding to SNPs of TG and HBP were included in enrichment analysis, and thereinto, genes relevant to TG were mainly enriched in lipid homeostasis and cholesterol metabolism, while genes concerned with HBP played their roles in regulation of cell growth, aldosterone synthesis and secretion and so forth.

**Conclusion:**

TG and HBP as risk factors were causally connected with IgAN, of which HBP was strongly related to the onset of IgAN, providing more reliable evidence for further exploring the relationship between TG and HBP and IgAN.

## 1 Introduction

Immunoglobulin A nephropathy (IgAN) is the most common primary glomerulonephritis in the world (1, 2) accounting for 40.5%–45% of all glomerular diseases in China (3). Patients can present with a range of symptoms, from haematuria or proteinuria to severe renal damage. The clinical course of most patients with IgAN is slow progression and sustained deterioration of renal function, and 30%–40% patients develop end-stage renal disease (ESRD) within 20 years after the first diagnosis (1). Even after renal transplantation, IgAN can frequently recur. Therefore, IgAN leads to a serious disease burden and it is necessary to explore this disease in depth.

In our cognition, IgAN is often accompanied by hyperlipidemia and high blood pressure (HBP), and we attribute these symptoms to renal dysfunction. However, recent researches show something different. A study shows that triglyceride (TG) is associated with renal interstitial fibrosis and is an independent risk factor (4). Some studies have analyzed the relationship between TG-glucose index and IgAN. The results show that high TG-glucose index is related to the high risk of renal dysfunction progress (5). Related literature also reports that HBP is an important factor that could accelerate the course of IgAN (6). These results indicate that both of TG and HBP have strong correlations with IgAN. However, it is still unclear whether there is a direct causal effect of TG and HBP on IgAN.

Mendelian randomization (MR) can be used to solve the causal hypothesis. It is a genetic tool variable analysis, which can evaluate the potential relationship between exposure and outcome (7). As MR uses instrumental variable analysis to simulate the randomization process of causal reasoning in randomized controlled trials, the design is less susceptible to confounding and reverse causal bias (8), which makes MR more and more widely used to explore whether there is a causal relationship between diseases and exposure factors.

Therefore, in this study, we take IgAN as the outcome, TG and HBP as the exposure factors, which aims to provide a new reference for the causal effect of TG and HBP on IgAN based on MR analysis.

## 2 Materials and methods

### 2.1 Data summary and pre-processing

Genome-wide association study (GWAS) summary data of IgAN and two exposure factors, TG and HBP, were downloaded from the GWAS Catalog^1^ and Integrative Epidemiology Unit (IEU) OpenGWAS^2^ databases, respectively. The dataset of IgAN (GCST90018866) was comprised of 653,143 samples (containing 477,784 European and 175,359 East Asian) and 20,059,328 single nucleotide polymorphisms (SNPs). The dataset ukb-d-30870_raw for TG contained 470,346 European samples and 13,586,007 SNPs. The dataset of HBP (ukb-a-437) was made up of 336,683 European samples and 10,894,596 SNPs. Whereafter, the “extract_instruments” function in R package “TwoSampleMR” (9) was employed to read exposure factors and screen instrumental variables (IVs), so as to ascertain the SNPs significantly correlated with exposure factors (*p* < 5 × 10^−8^) as IVs, followed by disposing of IVs with linkage disequilibrium (clump = TRUE; *r*^2^ = 0.001; kb = 10,000).

### 2.2 Mendelian randomization analysis

Firstly, the effect equipotential and effect size were harmonized through the “harmonise_data” function in “TwoSampleMR” for follow-up analysis. Five algorithms were employed to execute MR analysis, including MR Egger (10), weighted median (11), inverse variance weighted (IVW) (12), simple mode (9), and weighted mode (13). Parenthetically, the MR results mainly referred to IVW method, and the results were exhibited by scatter plot, forest plot and funnel plot. Finally, the reliability of MR results was assessed with the help of the sensitivity analysis, such as heterogeneity, horizontal pleiotropy and leave-one-out (LOO) analysis. Thereinto, if there was heterogeneity via Cochran’s *Q* test (*p* < 0.05), the random-effect IVW method was used to estimate the causal effect of TG and HBP on IgAN. The purpose of horizontal pleiotropy test was to evaluate the presence of confounding factors in MR analysis by “mr_pleiotropy_test” function. For LOO analysis, the meta effect of the remaining SNPs was calculated through the “mr_leaveoneout” function after stepwise elimination of each SNP. In the end, multivariable MR (MVMR) analysis was also performed to explore the effect on IgAN when TG and HBP existed simultaneously.

### 2.3 Functional enrichment and interaction analysis of genes related to SNPs

In order to explore the biological function and signal pathway of the relevant genes regulated by SNPs, the genes corresponding to SNPs related to TG and HBP were obtained with the help of Variant Effect Predictor in Ensembl database.^3^ Subsequently, the enrichment analysis of these genes was carried out via R package “clusterProfiler” (14) based on Gene Ontology (GO) and Kyoto Encyclopedia of Genes and Genomes (KEGG) databases (*p*_adj_ < 0.05). At last, the protein–protein interaction (PPI) network was constructed (interaction threshold = 0.7) to comprehend the interactions among proteins encoded by these gens through STRING database.^4^

## 3 Results

### 3.1 TG and HBP as risk factors were causally associated with IgAN

**Figure 1:**
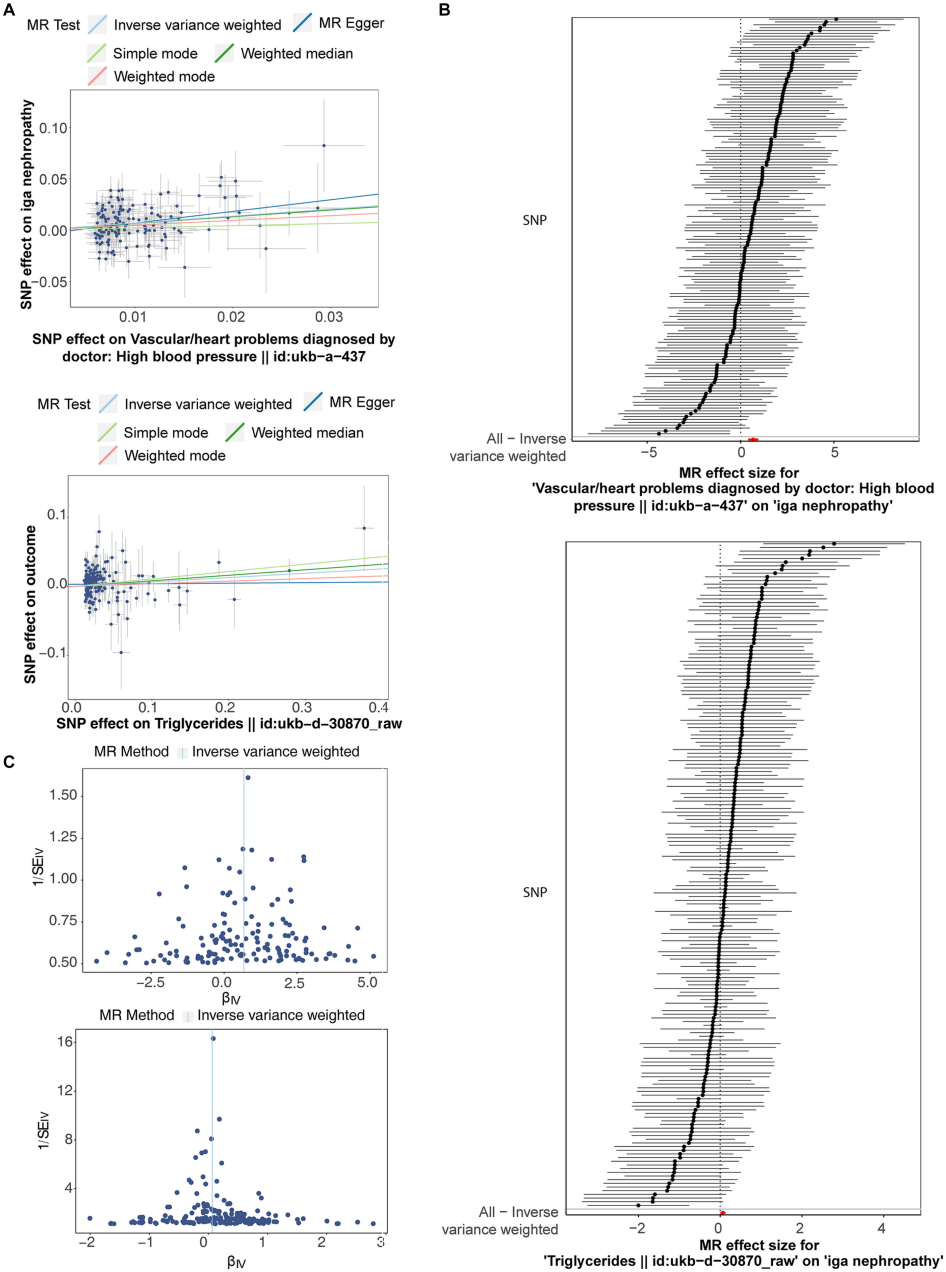
The scatter plots, forest plots and funnel plots related to Mendelian randomization (MR) analysis of triglyceride (TG) and high blood pressure (HBP) on immunoglobulin A nephropathy (IgAN). **(A)** Scatter plots indicated the tendencies of positive correlation of TG and HBP with IgAN in line with the MR results. **(B)** The forest plots showed that the overall effect of TG and HBP on IgAN based on IVW models was prominently hazardous. **(C)** Single nucleotide polymorphisms (SNPs) were symmetrically distributed along both sides of IVW line.

After filtrating, a total of 181 and 146 SNPs related to TG and HBP were acquired as IVs for univariate MR analysis, respectively (Supplementary Table S1). As presented in Table 1, the causal effects of TG (*p* = 0.046) and HBP (*p* = 7.09 × 10^−7^) on IgAN were discovered based on IVW method, and both TG [odds ratio (OR) = 1.065, 95% confidence interval (CI) = 1.001–1.133] and HBP (OR = 1.970, 95% CI = 1.507–2.575) were risk factors for IgAN. The scatter plots appeared the tendencies of positive correlation of TG and HBP with IgAN in line with the MR results (Figure 1A). The forest plots were drawn to evaluate the diagnostic performance of each SNP, showing that the overall effect of TG and HBP on IgAN based on IVW models was prominently hazardous (Figure 1B). The valid SNPs included in MR analysis were distributed symmetrically in the funnel plots, which conformed to Mendel’s second law random grouping (Figure 1C).

**Table 1:** Univariate Mendelian randomization (MR) analysis using various methods.

Exposure	Method	*p*-value	OR	95% LCI	95% UCI
TG	MR Egger	0.860178424	1.008013466	0.922468842	1.101491023
	Weighted median	0.114330759	1.083158067	0.980911369	1.196062595
	Inverse variance weighted	0.046415787	1.065168167	1.001001971	1.133447542
	Simple mode	0.355590693	1.112947764	0.887410032	1.39580654
	Weighted mode	0.341171249	1.038137991	0.961329326	1.121083546
HBP	MR Egger	0.00648	3.106927	1.390121	6.943996
	Weighted median	0.00047	1.913048	1.329942	2.751814
	Inverse variance weighted	7.09E-07	1.969895	1.506828	2.575267
	Simple mode	0.693659	1.231892	0.437305	3.47025
	Weighted mode	0.235121	1.593276	0.740844	3.426533

### 3.2 Reliability of the univariate MR results was illustrated by sensitivity analysis

**Figure 2:**
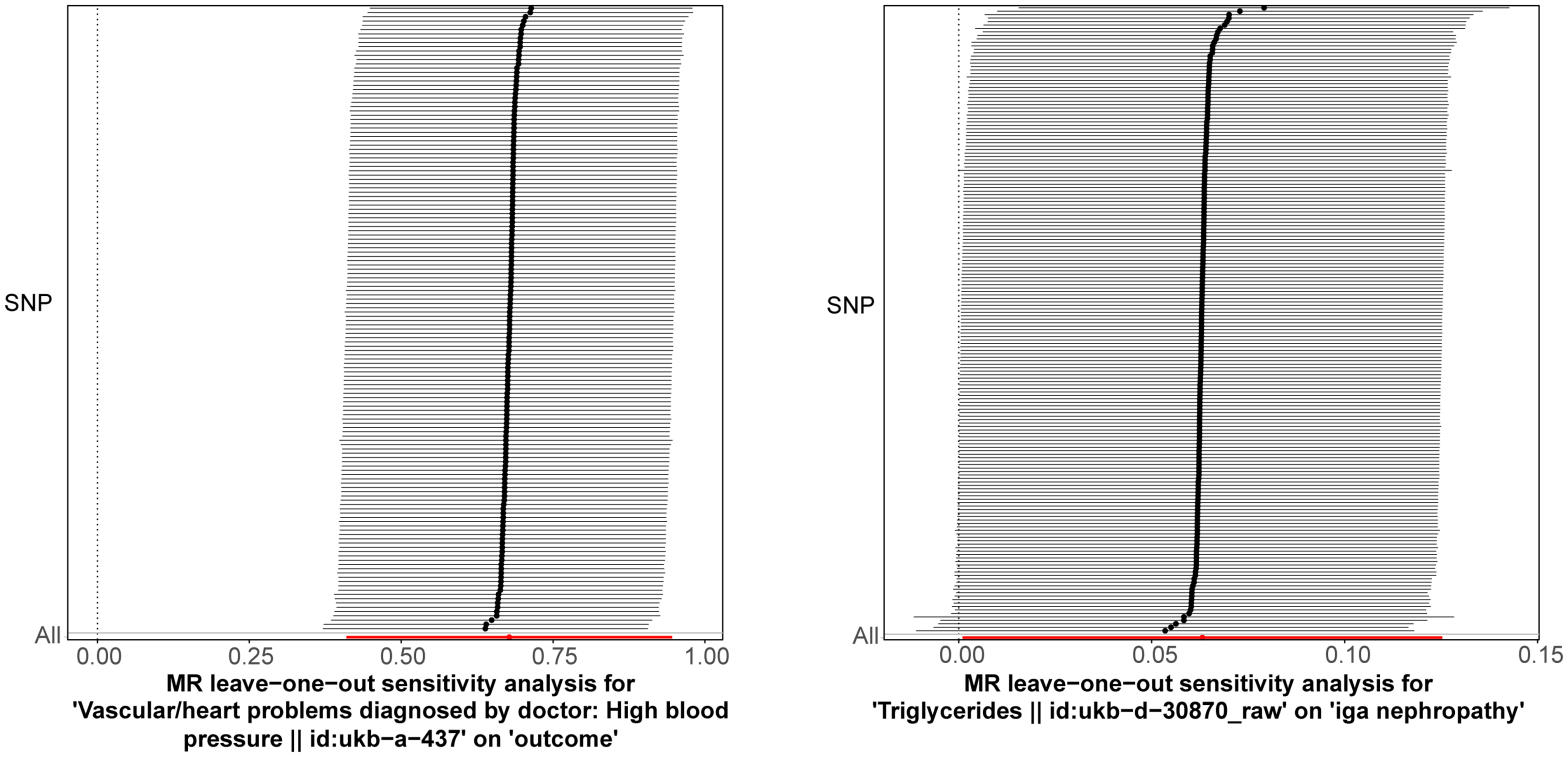
Leave-one-out method was used to examine if there was severe SNPs bias. The lines connected by black dots are smooth, and there are no serious biased points.

Following closely on the univariate MR analysis, the sensitivity analysis was proceeded to evaluate the reliability of MR results. There were significant heterogeneities for TG (*Q* = 231.488, *p* = 0.006) and HBP (*Q* = 186.553, *p* = 0.011) through Cochran’s *Q* test, thus, the random-effect IVW method was used to estimate their effect on IgAN (Table 2). Simultaneously, there were no horizontal pleiotropies for TG (Intercept = 0.00294; *p* = 0.091) and HBP (Intercept = −0.00456; *p* = 0.241) in the MR-Egger test, meaning there were no confounding factors (Table 3). Moreover, there was no SNPs of severe bias by LOO method, which also demonstrated the reliability of MR results (Figure 2). In conclusion, TG and HBP were risk factors in causal connection with IgAN.

**Table 2:** Cochran’s *Q* test for heterogeneities test.

Exposure	Method	*Q*	*Q*_*p*-value
TG	MR Egger	227.8110938	0.007950183
	Inverse variance weighted	231.4879518	0.005778428
HBP	MR Egger	184.7733929	0.012363195
	Inverse variance weighted	186.5526929	0.011367658

**Table 3:** MR-Egger test for examining the horizontal pleiotropy.

Exposure	Egger_intercept	SE	*p*-value
TG	0.002935745	0.001727193	0.090920514
HBP	−0.004561132	0.003873348	0.240911298

### 3.3 HBP was the direct influencing factor on IgAN via MVMR analysis

**Figure 3:**
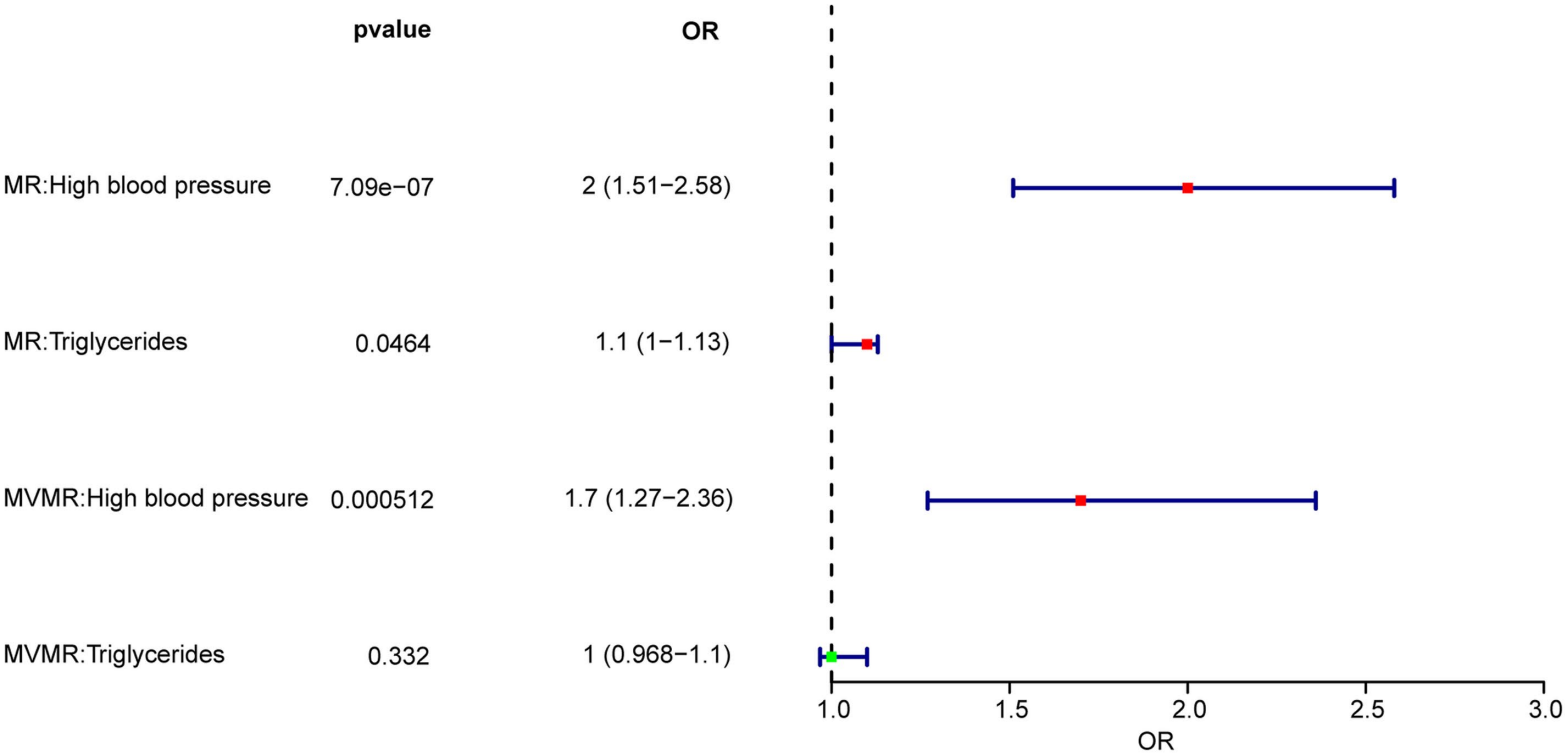
Univariate and multivariable MR (MVMR) analysis of TG and HBP on IgAN.

After the screening stage, a total of 238 independent SNPs related to TG and HBP were identified as IVs for MVMR analysis, whose characteristics of genetic variants associated were presented in Supplementary Table S2. With respect to the results of MVMR analysis, TG (OR = 1.032, 95% CI = 0.968–1.101) and HBP (OR = 1.731, 95% CI = 1.270–2.360) were risk factors for IgAN, nevertheless, only HBP was causally related to IgAN (*p* = 0.000512) (Figure 3 and Table 4). In short, HBP had the directly causally influence on IgAN when HBP and TG occurred at the same time.

**Table 4:** The results of multivariable MR (MVMR) analysis.

Exposure	OR	95% LCI	95% UCI	*p*-value
TG	1.731219273	1.270225845	2.359517548	0.000512514
HBP	1.03233037	0.968005929	1.1009292	0.332364246

### 3.4 Enrichment analysis of genes corresponding to SNPs of TG and HBP

**Figure 4:**
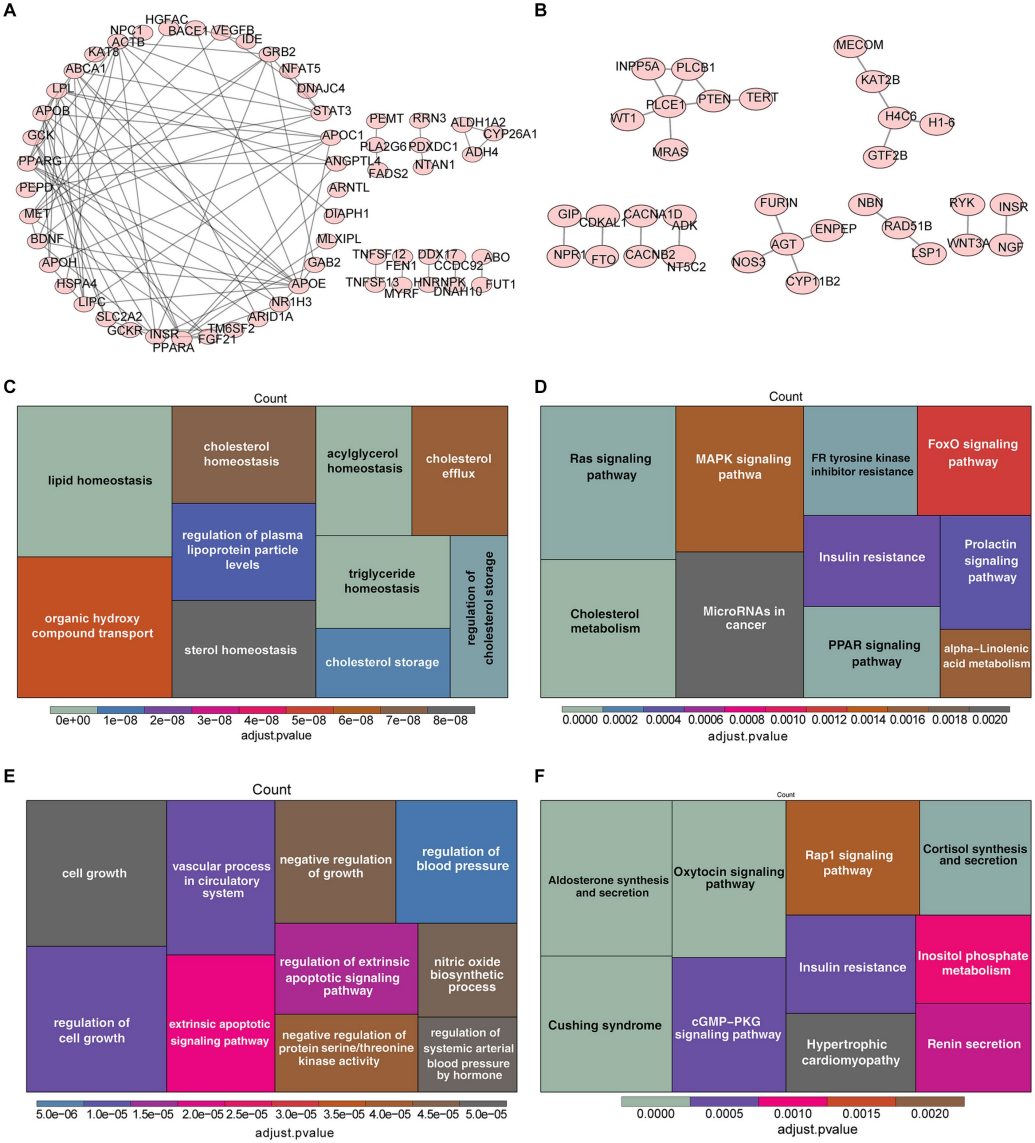
Enrichment analysis of genes corresponding to SNPs of TG and HBP. **(A)** Protein–protein interactions (PPI) network of 56 genes related to SNPs of TG. **(B)** PPI network of 32 genes correlated with SNPs of HBP. The Gene Ontology (GO) analysis **(C)** and the most enriched Kyoto Encyclopedia of Genes and Genomes (KEGG) analysis **(D)** of 208 genes corresponding to SNPs of TG. Functional enrichment analysis of 153 genes corresponding to SNPs of HBP, **(E)** GO, **(F)** KEGG.

For the sake of comprehending the biological functions and pathways involved in genes corresponding to SNPs, the enrichment analysis was carried out for 208 genes related to TG and 153 genes correlated with HBP (Supplementary Table S3), of which 56 genes for TG (such as APOC1, STAT3, PPARG) and 32 genes for HBP (such as PLCE1, AGT, H4C6) were incorporated into the PPI network (Figures 4A,B). On the part of GO enrichment analysis for 208 genes corresponding to SNPs of TG, a total of 184 GO items were sought out, including 162 items in biological process (BP), 7 items in cellular components (CC) and 15 items in molecular functions (MF), such as lipid homeostasis, organic hydroxy compound transport and regulation of plasma lipoprotein particle levels (Figure 4C and Supplementary Table S4). Moreover, these genes were markedly enriched in 12 KEGG pathways, including Ras signaling pathway, cholesterol metabolism, MAPK signaling pathway and so on (Figure 4D and Supplementary Table S5). In regard to the enrichment analysis for 153 genes corresponding to SNPs of HBP, they were enriched in 117 GO items (116 BP and 1 CC items) and 16 KEGG pathways (Figures 4E,F and Supplementary Tables S6, S7), for instance, regulation of cell growth, negative regulation of growth and extrinsic apoptotic signaling pathway in GO; aldosterone synthesis and secretion, oxytocin signaling pathway and Cushing syndrome in KEGG.

## 4 Discussion

IgAN is a common kidney disease with long course, which brings serious physical and mental damage and economic burden to patients (1). Elevated TG and HBP are common in IgAN, and studies have also shown that they are related to disease progression (4, 6). Here, our study takes TG and HBP as the exposure factors and IgAN as the outcome, and explores their direct causal relationship by MR analysis. The result of univariate MR analysis shows that both TG and HBP are the causes of IgAN based on IVW method. When both of them exist at the same time, multivariate MR analysis suggests that HBP plays a more significant role in IgAN. Due the randomization variables are genetic variants, MR studies can evaluate the relationships discovered by clinical observational studies and reasonably avoid reverse causation (8). Relatively, most of the clinical researches are limited in small-sample or single-centered and can not avoid potential comorbid confounders. This is the strength of MR. Furthermore, to judge the reliability of the analysis results, sensitivity analysis was carried out. The result shows that there were no confounding factors and no SNPs of severe bias in MR analysis, which ensures the reliability of causality between TG, HBP and IgAN.

The pathophysiological mechanism of IgAN caused by TG and HBP is still unclear. Previous studies have shown that the pathogenesis of IgAN may be related to abnormal glycosylation and deposition of IgA (1). Our results show that TG and HBP can cause IgAN, and one possible explanation is that TG and HBP damage the vascular endothelial structure, which is more likely to lead to the deposition of IgA, thereby activating the follow-up pathophysiological processes such as complement response and inflammatory factor release. In cardiovascular diseases, it is generally believed that the increase of TG is accompanied by abnormal lipoprotein metabolism, which can cause lipid deposition and further promote the occurrence of atherosclerosis (15). In addition to promoting atherosclerosis, lipids have toxicity and can also directly damage global cells and promote podocyte apoptosis (16). Moreover, elevated TG is often accompanied by decreased high-density lipoprotein, which contributes to endothelial dysfunction, accelerated atherosclerosis, oxidative stress, and systemic inflammation in patients with chronic kidney disease (CKD) (17). For renal interstitium, it has been reported that TG is related to tubular atrophy and interstitial fibrosis, and is an independent risk factor (4). However, some studies have pointed out that TG is not an independent risk factor although it is related to renal tubular atrophy and interstitial fibrosis (18). Other studies have pointed out that TG has nothing to do with tubular atrophy and interstitial fibrosis (19). Generally speaking, TG may contribute to glomerular vascular injury, but it is controversial in the pathophysiological process of renal interstitial. Similarly, HBP can also damage the vascular structure in many ways, such as stress shock, vascular remodeling, inflammatory reaction and so on, which can accelerate the progression of IgAN and affect the prognosis of IgAN (20). For example, HBP may cause damage of normal renal vascular structure, leading to glomerular hypertrophy and renal tubulointerstitial fibrosis in IgAN patients (21). The enlargement of glomerulus increases the tensile stress on the podocyte foot process covering capillaries, which leads to podocyte damage, loss of glomerular barrier function, proteinuria, glomerulosclerosis and gradual loss of renal function. At the same time, vascular thickening and hyalinization caused by HBP can lead to interstitial ischemia and hypoxia, which aggravates the fibrosis process (22). Eventually, the disease process is accelerated, renal function damage and symptoms are obvious, prompting patients and doctors to seek pathological diagnosis and making the diagnosis and discovery rate of IgAN higher.

In order to better understand the possible molecular mechanism of TG and HBP on IgAN, we annotated SNP used in univariate MR analysis as gene symbol for enrichment analysis. In the univariate analysis of TG, GO was enriched to 184 items, including 7 CC, 15 MF and 162 BP. In KEGG, 12 functional pathways were enriched. In univariate analysis of HBP, GO was enriched to 117 items, including 1 CC and 116 BP. In KEGG, 16 functional pathways were enriched. Some of these potential mechanisms mentioned in our enrichment analysis have also been confirmed in recent articles. For example, the study believes that when lipid homeostasis is broken, cholesterol and TG are dysregulated in podocytes, endothelial and tubular cells, and contribute to CKD progression (23). Accumulation of fatty acids triggers mitochondrial and kidney cell damage by promoting cellular sterile inflammation via innate immune system activation and fibrosis via PPAR–FAO (fatty acids oxidation) axis (23). There is also a study showing that maintaining lipid homeostasis is a new therapeutic target for acute kidney injury (AKI) and can prevent AKI from transforming into CKD by blocking the damage of lipid accumulation to renal tubular epithelial cells (24). Another research indicates that apolipoprotein C1 (APOC1) is mainly expressed in renal tubular epithelial cells and its expression is elevated in IgAN serum. APOC1 can promote HK-2 cell fibrosis by activating the NF-κB signaling pathway (25). On the other hand, aldosterone synthesis and secretion, regulation of cell growth, negative regulation of growth and extrinsic apoptotic signaling pathway are involved in HBP. Aldosterone is mainly related to renin angiotensin system, which can cause HBP in patients with IgAN, meanwhile renin angiotensin system blockades are also the recommended IgAN treatment scheme in the guide, which can delay the progress of IgA nephropathy (26), including what we know angiotensin-converting-enzyme-inhibitors and angiotensin-II receptor blockers. *In vitro* studies, aldosterone is mediator for glomerulotubular crosstalk, which induces apoptosis of proximal tubular epithelial cells through nicotinamide adenine dinucleotide phosphate dependent generation of reactive oxygen species (27). Phospholipase C-ε1 (PLCE1) can regulate cell growth, proliferation, differentiation, migration and apoptosis after being activated. Loss-of-function mutations in PLCE1 have been detected in patients with nephrotic syndrome (28). Compared with wild-type littermates, HBP increased proteinuria by 20 times and glomerulosclerosis significantly in PLCE1 deficient mice (29). Furthermore, PLCE1-deficient mice was demonstrated with diffuse mesangial expansion, podocyte loss, and focal podocyte foot process effacement in kidney (29). Angiotensinogen (AGT) is the key point of RAS system, and AGT levels influence RAS activation. Related to glomerular and renal tubular injury, AGT has the highest correlation with the degree of renal fibrosis in IgAN patients (30). The possible mechanism is that AGT can increase inflammatory factors and reactive oxidation factors via angiotensin II, induce the formation of crescent in renal tissue and aggravate the degree of renal fibrosis (30). In short, many protein, physiological processes and pathways in enrichment analysis have been confirmed in recent studies, which are related to the occurrence and progress of nephropathy. But to our knowledge, many genes and pathways enriched and analyzed by us have not been studied in IgAN yet. There still remains a broad research space for an investigation into the mechanism in the future.

There is also limitation of the MR analysis. There are multiple influencing factors in the complex disease of IgAN, not all of which were considered in this study, and this will be the direction of future research we will conduct. As we know, the incidence of HBP is related to age and sex, which is more common in men, while the prevalence rate of women shows an upward trend with age (31). In addition, the statistical analysis in the study (32) suggests that the proportion of TG in men is lower than that in women, and it is on the rise with the increase of age. At the same time, research identifies men as a risk factor for the poor outcome of IgAN, and the pathological characteristics of men and women are also different (33). To sum up, gender and age may affect the exposure factors and outcome in this study. Although we try to reduce the influence of confounding factors on the research results by setting the screening threshold of SNPs and a series of sensitivity analysis, completely ruling it out is difficult. Therefore, we will collect a large number of samples and data sets to further verify the influence of factors such as age and gender on the causal relationship between TG, HBP and IgAN. In addition, the studied population of TG and HBP is European, but population of IgAN is mixed (73.15% European and 26.85% East Asian). In this analysis, we did not eliminate the East Asian samples in IgAN, because we could not download the character data of the data set and could not eliminate the samples. Considering the different incidence of IgAN between Europeans and East Asians, this limitation may affect our analysis performance. MR study on the classification of different races is needed in the future. In addition, the GWAS Catalog database makes no mention of diagnostic criteria for HBP, which would also be of concern to us.

## 5 Conclusion

In conclusion, there is a causal effect of TG and HBP on IgAN, both of them are risk factors, and HBP is the direct influencing factor of IgAN. Since the sample data used in this study originated in Europe and East Asia, the causality currently applies only to these regions. Enrichment analysis of genes corresponding to SNPs of TG and HBP might spark interest in further molecular biological mechanism investigation in IgAN.

## Data availability statement

The datasets presented in this study can be found in online repositories. The names of the repository/repositories and accession number(s) can be found in the article/Supplementary material.

## Ethics statement

Ethical approval was not required for the study involving humans in accordance with the local legislation and institutional requirements. Written informed consent to participate in this study was not required from the participants or the participants’ legal guardians/next of kin in accordance with the national legislation and the institutional requirements.

## Author contributions

YY: Writing – original draft, Formal analysis, Methodology, Software. YL: Formal analysis, Software, Writing – review & editing. XF: Validation, Writing – original draft. CD: Data curation, Validation, Writing – review & editing. JZ: Data curation, Writing – original draft. ZL: Conceptualization, Data curation, Investigation, Supervision, Writing – review & editing.

## Footnotes

^1^ https://www.ebi.ac.uk/gwas/

^2^ https://gwas.mrcieu.ac.uk/

^3^ https://asia.ensembl.org/Tools/VEP

^4^ https://cn.string-db.org/
